# Effect of Food and an Animal’s Sex on P-Glycoprotein Expression and Luminal Fluids in the Gastrointestinal Tract of Wistar Rats

**DOI:** 10.3390/pharmaceutics12040296

**Published:** 2020-03-25

**Authors:** Liu Dou, Francesca K. H. Gavins, Yang Mai, Christine M. Madla, Farhan Taherali, Mine Orlu, Sudaxshina Murdan, Abdul W. Basit

**Affiliations:** 1 UCL School of Pharmacy, University College London, 29–39 Brunswick Square, London WC1N 1AX, UK; liu.dou.14@ucl.ac.uk (L.D.); francesca.gavins.13@ucl.ac.uk (F.K.H.G.); christine.madla.16@ucl.ac.uk (C.M.M.); farhan.taherali.15@ucl.ac.uk (F.T.); m.orlu@ucl.ac.uk (M.O.); s.murdan@ucl.ac.uk (S.M.); 2 School of Pharmaceutical Sciences (Shenzhen), Sun Yat-sen University, Guangzhou 510275, China; maiy6@mail.sysu.edu.cn

**Keywords:** P-glycoprotein, multidrug-resistant protein 1 (mdr1), ABCB1, preclinical development, sex differences, food effect, oral drug delivery, liquid chromatography tandem mass spectrometry (LC-MS/MS)

## Abstract

The rat is one of the most commonly used animal models in pre-clinical studies. Limited information between the sexes and the effect of food consumption on the gastrointestinal (GI) physiology, however, is acknowledged or understood. This study aimed to investigate the potential sex differences and effect of food intake on the intestinal luminal fluid and the efflux membrane transporter P-glycoprotein (P-gp) along the intestinal tract of male and female Wistar rats. To characterise the intestinal luminal fluids, pH, surface tension, buffer capacity and osmolality were measured. Absolute P-gp expression along the intestinal tract was quantified via liquid chromatography-tandem mass spectrometry (LC-MS/MS). In general, the characteristics of the luminal fluids were similar in male and female rats along the GI tract. In fasted male rats, the absolute P-gp expression gradually increased from the duodenum to ileum but decreased in the colon. A significant sex difference (*p* < 0.05) was identified in the jejunum where P-gp expression in males was 83% higher than in females. Similarly, ileal P-gp expression in male rats was approximately 58% higher than that of their female counterparts. Conversely, following food intake, a significant sex difference (*p* < 0.05) in P-gp expression was found but in a contrasting trend. Fed female rats expressed much higher P-gp levels than male rats with an increase of 77% and 34% in the jejunum and ileum, respectively. A deeper understanding of the effects of sex and food intake on the absorption of P-gp substrates can lead to an improved translation from pre-clinical animal studies into human pharmacokinetic studies.

## 1. Introduction

Since 2002, the United States Food and Drug Administration (FDA) has recommended that food-effect bioavailability studies should be conducted in early drug development research for the understanding of possible food–drug interactions [1,2]. In the gastrointestinal (GI) tract, food and its subsequent digestion products can affect drug absorption by changing the luminal fluid environment itself or by physically or chemically interacting with the drug [3]. In addition to food, drug pharmacokinetics can be significantly affected by sex, as numerous studies have shown that males and females can respond differently to medicines [4]. A prominent example is digoxin, a P-glycoprotein (P-gp) substrate, which has shown higher mortality rates among women patients when compared with males for the treatment of heart failure [5]. Over 25 years ago, the FDA issued a guideline for industry titled “Study and Evaluation of Gender Differences in the Clinical Evaluation of Drugs” requiring the evaluation of both sexes in all phases of drug clinical trials, including early safety efficacy and dose-determination studies [6,7]. 

Whilst the evaluation of sex differences has been well-implemented in human clinical trials, there is no consistent consensus to reform pre-clinical animal studies [8]. Pre-clinical studies have traditionally focused on male-biased experiments with the absence of female animals. As such, there is a lack of evidence-based findings in female animal models [9,10]. Incomplete pharmacokinetic data interpretation from pre-clinical to clinical studies may potentially obscure key sex differences. Only in recent years has the impact of overlooking sex differences in pre-clinical studies been realised. In December 2013, the European Commission required applicants of the Horizon 2020 research program to include sex analyses in their projects [11]. Furthermore, the United States National Institute of Health (NIH) requires applicants to incorporate a sex-balanced group of participants in pre-clinical research [8,10]. However, to date, no such practice has been followed, and studies continue to neglect the need for sex-based evaluation in pre-clinical studies [12,13].

Rats are one of the most commonly used pre-clinical animal models in oral drug development due to their low cost, ease of handling, and, more importantly, the similarities between their GI tract and those of humans [14,15,16,17]. Our group has previously found a sex difference in the bioavailability of a P-gp substrate ranitidine after administration with the excipient poly(ethylene glycol) (PEG) 400 in humans [18] and rats [19,20]. Furthermore, the authors have previously found that food consumption resulted in a change in the relative P-gp expression to different extents in male and female rats [21]. The efflux transporter P-gp serves as an essential protective mechanism in the intestinal luminal environment by heavily mediating oral drug absorption. In addition, P-gp has been reported as a significant contributor to sex differences in pharmacokinetic responses [22]. The underlying mechanism of this sex-based phenomenon, however, is still not fully understood.

Conflicting reports exist on the P-gp expression levels along the GI tract. MacLean et al. reported that the intestinal abundance of P-gp increased from the proximal to distal regions of the intestine with no sex differences observed [23], which is in contrast to our findings that display a significant sex difference [21]. However, Western blot techniques were used for P-gp quantification. Limitations of such a technique include the reliance on an internal reference protein and multi-step analyses, which can produce variable results. In contrast, the present study uses liquid chromatography-tandem mass spectrometry (LC-MS/MS)-based targeted proteomics to provide comprehensive absolute P-gp expression data.

The absorption of an orally dosed product requires the drug first to undergo dissolution where the solubility into the surrounding GI luminal fluids is the rate-limiting step. Previous studies have highlighted a significant sex difference in rat intestinal luminal fluids. However, this was only investigated under fed-state conditions [19], and food consumption can elicit unpredictable changes in the luminal fluid. Differences in luminal fluids properties and subsequent effects on drug dissolution or absorption processes could cause differences in bioavailability between the sexes. A better understanding of the GI physiology of male and female rats in fasted and fed states is important for the accurate interpretation of sex differences in oral drug behaviour in humans. Therefore, this study aims to investigate the potential interaction between food intake and sex in the intestinal environment by the comprehensive characterisation of GI luminal fluids and absolute P-gp expression in male and female rats.

## 2. Materials and Methods 

### 2.1. Reagents and Materials 

HPLC-grade water, methanol, peroxide-free tetrahydrofuran and trifluoroacetic acid were purchased from Fisher Scientific (Loughborough, UK) for luminal fluids characterisation. NaOH and HCl (0.1 M standards) were used for buffer capacity determinations and were procured from Sigma–Aldrich (Dorset, UK). The standard peptide for quantification of P-gp and its stable isotope-labelled internal standard were of analytical grade (purity > 95%) and were synthesised and quantified using the amino acid analysis by Sigma AQUA peptides service (Poole, Dorset, UK). Iodoacetamide, dithiothreitol (CAS: 3483-12-3), MS-Grade trypsin (CAS: 9002-07-7) and formic acid (CAS: 64-18-6) were bought from Thermo Fisher (Gloucester, UK). Ammonium bicarbonate BioUltra, chloroform, as well as the LC-MS grade acetonitrile and water were obtained from Sigma (Dorset, UK). All other chemicals and kits are noted individually in the following methods.

### 2.2. Experimental Animals

Twelve male and 12 female Wistar rats (8 weeks old, approximately 250 g and 200 g, respectively) were purchased from Harlan UK Ltd. (Bicester, UK). The rats were housed at room temperature (25 °C) and in a light-dark cycle of 12 h. Food (EURodent Diet 22%, LabDiet, St. Louis, MO, USA) and tap water were provided ad libitum, and the rats were allowed to acclimatise for at least 7 days. To investigate the fasted state, an overnight fasting (12 h) was applied for 6 male and 6 female rats prior to the experiments the following morning at 8 am. For the fed state, 6 female and 6 male rats were fed ad libitum, and the experiments began in the morning at 8 am. All procedures were approved by the Home Office (PPL No. 70/6421) and were conducted in accordance with the Animals (Scientific Procedures) Act 1986, UK.

### 2.3. Characterisation of Luminal Fluids in the GI Tract

The rats were sacrificed by CO_2_ asphyxiation in the morning of the experiment. All measurements were performed on the supernatant obtained from the GI fluids of the laboratory rats, except for the pH measurements. 

The pH of the GI tract was measured in situ using a pH meter equipped with an FC202 electrode, designed for measurements in viscous and semi-solid materials (HI99161, Hanna Instruments, Leighton Buzzard, UK). pH was determined by introducing the pH probe into the opening created by sectioning parts of the GI tract (stomach (fundus and antrum), duodenum, jejunum, ileum, caecum and colon). For each GI segment, two in situ measurements were performed, one at the proximal opening (A) and the second at the distal end (B). The stomach and entire intestinal tract contents were then promptly extracted and separated into the duodenum, jejunum, ileum, caecum and colon within 10 min. The sections were placed into 1.5 mL Eppendorf tubes and centrifuged at 13000 rpm for 20 min (Centrifuge 5415D, Eppendorf AG, Hamburg, Germany). The supernatant obtained was kept at −80 °C until analysis using the following characterisation techniques.

Osmolality was measured using a Digital Micro-Osmometer (Type 5R) (Hermann Roebling MESSTECHNIK, Berlin, Germany). Surface tension was measured using a Delta 8 Tensiometer (Kibron Inc, Helsinki, Finland) controlled by Delta-8 manager software (version 3.8). The measurement was performed using a DynePlates (96-well plate designed for tensiometer) with 50 µL of the sample in each well. 

Buffer capacity was measured at pH changes of 0.5 and 1.0 units by adding aliquots (10 µL) of 0.1 M HCl (for intestinal fluids) or 0.1 M NaOH (for gastric fluids) to a 300 µL supernatant pooled sample from GI fluid to achieve the desired pH change. Buffer capacity was then calculated using the following Equation (1):

(1) $$\beta \;\left(mmol/L/\Delta \mathrm{pH}\right)=\frac{M_{a}\times V_{a}}{\Delta pH}\times \frac{1000}{V_{b}}$$

where β is the buffer capacity, $M_{a}$ is the molarity of the acid, $V_{a}$ is the volume of acid in mL, $V_{b}$ is the volume of buffer in mL and ΔpH is the change in pH unit. 

Due to the small amount of fluids available in some of the intestinal segments, the measurements were conducted using pooled samples, where fluids from the same segment of different rats were mixed to increase the available volume.

### 2.4. Absolute Quantification of P-glycoprotein 

#### 2.4.1. Tissue Preparation

On the day of the experiment, the rats were sacrificed in a CO_2_ euthanasia chamber (Schedule 1 method). Tissues from the stomach and all segments of the intestine were rapidly removed and kept in an ice-bath filled with Krebs-Bicarbonate Ringer’s solution (KBR) at pH 7.4. The KBR solution consisted of 10 mM d-glucose, 1.2 mM calcium chloride (CaCl_2_), 1.2 mM magnesium chloride (MgCl_2_), 115 mM sodium chloride (NaCl), 25 mM sodium bicarbonate (NaHCO_3_), 0.4 mM monopotassium phosphate (KH_2_PO_4_) and 2.4 mM dipotassium phosphate (K_2_HPO_4_) (Clarke, L.L, 2009). A pH of 7.4 was achieved and adjusted using sodium hydroxide/hydrochloric acid. The tissue was stored in a beaker of ice-cold KBR for approximately 20 min to acclimatise to a low temperature to minimise potential tissue damage during preparation. 

Subsequently, 1 cm pieces of the following segments were excised; glandular side of the stomach, mid part of the duodenum, proximal jejunum, distal to mid ileum and descending colon. The mucosal tissue was obtained by opening the tissue along the mesenteric border and gently squeezing the serosal side of tissue with a coverslip on top of an ice-cold glass plate. The prepared tissue was then immediately used in the following methods.

#### 2.4.2. Total Protein Extraction 

The prepared tissue was placed in a glass vial containing 3 mL of freshly prepared lysis buffer (50 mM Tris, 250 mM NaCl, 5 mM EDTA, 1 mM Na_3_VO_4_, 1 mM PMSF, 1% Nonidet P40 and a protease inhibitor cocktail), and homogenised for 20 s at 10,000 rpm with a T18 digital ULTRA-TURRAX^®^ (IKA, Oxford, UK). The homogenised tissue solution was then incubated at 4 °C for 2 h for protein extraction. The solution was then transferred to a 1.5 mL Eppendorf tube and centrifuged at 10,000 rpm at 4 °C for 10 min. The supernatant was transferred to micro-tubes and stored at −20 °C. The total protein concentration was determined using the Pierce BCA Protein Assay Kit (ThermoFisher, Gloucester, UK). 

#### 2.4.3. Protein Sample Digestion 

Protein samples of a concentration of 50 μg were adjusted to 200 μl protein solution with 50 mM ammonium bicarbonate buffer and then mixed with 4 μl of freshly prepared dithiothreitol (20 mM). The solution was then incubated for 20 min at 56 °C for the protein denaturation step. After cooling, the alkylation step was carried by adding 8 μl iodoacetamide (375 mM) and incubated for 20 min at 37 °C in a dark environment. Then the precipitation step was carried out by adding 600 μl cold methanol and 150 μl cold chloroform to the sample solution. The tube was inverted several times, 450 μl cold water was added, and then the sample was immediately centrifuged at 15,000 rpm for 5 min at 4 °C. After centrifugation, the upper layer (above the suspended protein pellet) was removed, and an additional 450 μl cold methanol was added, and then the tube was inverted to wash the protein pellet. The sample was then centrifuged again at 15,000 rpm for 5 min at 4 °C. Immediately after centrifugation, the supernatant was removed, and the sample was centrifuged at 15,000 rpm for 1 min at 4 °C. The supernatant was then removed fully. 47 μl of ammonium bicarbonate buffer (50 mM) was added to the precipitated protein pellet and the protein was re-suspended by applying intermittent sonication. 5 μl of trypsin (0.5 μg/μl) was added to the re-suspended protein solution and was incubated for 4 h at 37 °C. The digestion process was stopped by adding 3 μl 50% formic acid in water, and 5 μl stable isotope-labelled internal standard (200 fmol/μl) was then added. The final processed sample solution (60 μl) was then centrifuged at 15,000 rpm for 5 min at 4 °C, and 30 μl of supernatant was then obtained for LC-MS/MS analysis. All sample digestion procedure steps were processed using Protein Lobind tubes (Eppendorf, Hamburg, Germany). 

#### 2.4.4. LC-MS/MS Analysis

The P-gp proteotypic peptide and its three multiple reaction monitoring (MRM) transitions have been selected from a previous study, as shown in Table 1 [24]. Calibration curves were established for all three transitions, and the P-gp absolute quantification was obtained from the mean values of the three calibration curves data. An Agilent 6460 triple quadrupole LC and mass spectrometer system coupled with Agilent Jet Stream technology was used for the analysis (Agilent Technologies, Santa Clara, CA, USA). A gradient elution was applied using a Kinetex C18 column (100 × 3.0 mm, 2.6 μm, Phenomenex, Torrance, CA, USA). The mobile phases were 0.1 % formic acid in water (solvent A) and 0.1% formic acid in acetonitrile (solvent B) with a flow rate of 0.5 mL/min. The gradient elution procedure started with 98% solvent A for 5 min and then a linear gradient from 98% solvent A to 75% solvent A over 10 min, held at 75% solvent A for 1 min and then changed to 55% solvent A for an extra 2 min. Solvent A was then changed back to 98% for 7 min until the end of the analysis. The mass spectrometer was equipped with electrospray ionisation and operated in the positive ion mode to monitor the three m/z transitions with 300 °C source temperature, nebuliser 45 psi, 11 L/min sheath gas flow, 500 V nozzle voltage, 20 collision energy as well as 7 cell accelerator voltage. All samples were analysed in duplicate, and the chromatograms were assessed with MassHunter Workstation software (Qualitative Analysis version B.06.00). 

### 2.5. Statistical Analysis

The results generated in the study were expressed as mean ± standard deviation (SD) (*n* = 6). The data were analysed by a one-way analysis of variance (ANOVA) in each segment, followed by a Tukey post-hoc analysis with a 95% confidence interval using IBM SPSS Statistics 16 (SPSS Inc., Chicago, IL, USA). 

## 3. Results

### 3.1. Sex Differences in Luminal Fluids Along the Rat GI Tract

#### 3.1.1. Gastrointestinal Fluid pH

**Figure 1:**
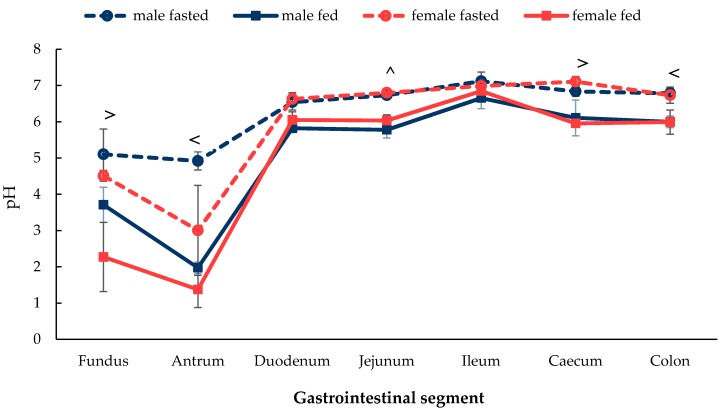
pH change in the luminal environment along the GI tract in male and female in the fasted state and fed state rats measured in situ. (Mean ± SD, *n* = 6). The following symbols (i) < denotes a statistical significance (*p* < 0.05) between fasted and fed state in male rats, (ii) > denotes a statistical significance (*p* < 0.05) between fasted and fed state in female rats and (iii) ^ denotes a statistical significance (*p* < 0.05) between fasted and fed state in both male and female rats.

As shown in Figure 1, there was no observed sex difference in the in situ pH along the stomach and intestinal segments of the GI tract in both fasted and fed male and female Wistar rats (*p* > 0.05). There was, however, an observable difference between the fasted and fed states in both sexes. To be precise, a significant difference in (i) the female rats between the fasted and fed states in the fundus and caecum, (ii) males rats between the fasted and fed states in the antrum and colon and (iii) in both sexes between the fasted and fed states in the jejunum (*p* < 0.05). No differences between the fasted and fed states in both sexes were observed in the duodenum and ileum (*p* > 0.05). For all groups, the stomach displayed the lowest pH in the glandular stomach segment (antrum), showing a lower pH than the forestomach (fundus). The stomach in the fed state showed a lower pH than the fasted state. Animal housing food and water were freely accessible for rats in the fed state, and following sacrifice, different volumes of food boluses were found in the GI tracts of the different rats. A sharp rise in pH was then observed from the antrum to the duodenum, which remained stable until the colon.

#### 3.1.2. Gastrointestinal Fluid Buffer Capacity

**Figure 2:**
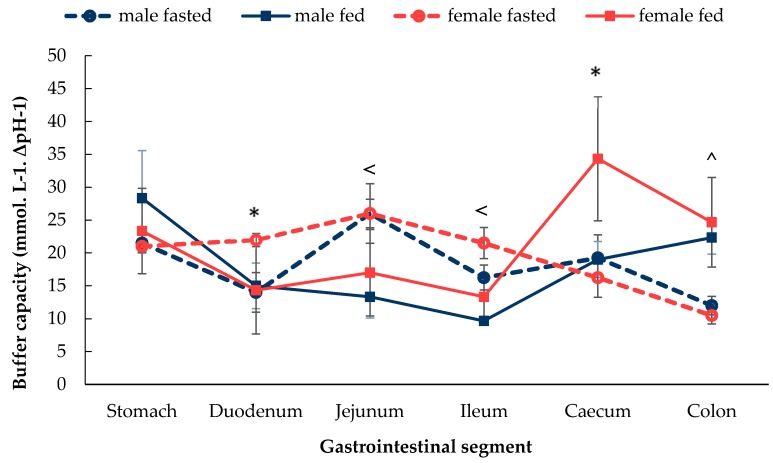
Buffer capacity (ΔpH = 1.0) of pooled fluids of sections of the GI tract in male and female in the fasted state and fed state rats. (Mean ± SD, *n* = 6). The values are the mean of several pooled fluids measurements. The following symbols (i) < denotes a statistical significance (*p* < 0.05) between fasted and fed state in male rats, (ii) ^ denotes a statistical significance (*p* < 0.05) between fasted and fed state in both male and female rats and (iii) * denotes a statistical significance (*p* < 0.05) between males and females.

As shown in Figure 2, the profiles of buffer capacity were similar between the groups. In fasted female rats, the buffer capacity was found to increase from the stomach to the small intestine and achieved the highest value in the jejunum. After the jejunum, the buffer capacity then exhibited a continual downward trend and reached the lowest value in the colon. In the fed female rats, however, the buffer capacity displayed a dynamic trend where it decreased from the stomach to duodenum, then increased in the jejunum, then decreased in the ileum. A sharp increase was then seen in the caecum, which then decreased in the colon. 

Fasted male rats displayed a fluctuating buffer capacity along the GI tract. In the fed male rats, the buffer capacity decreased from the stomach to the small intestine, then increased from the ileum to the colon. Significant differences between the fasted and fed states were seen (i) in the jejunum and ileum of male rats and (ii) in the colon of male and female rats.

A significant sex difference was noticed in the fasted duodenum, where buffer capacity was 14 ± 3 mmol. L^−1^. ΔpH^−1^ in males and 22 ± 1 mmol. L^−1^. ΔpH^−1^ in females. Furthermore, a significant sex difference was noticed in the fed caecum, where the female rat buffer capacity was 1.8-fold higher than in male rats.

#### 3.1.3. Gastrointestinal Fluid Osmolality

**Figure 3:**
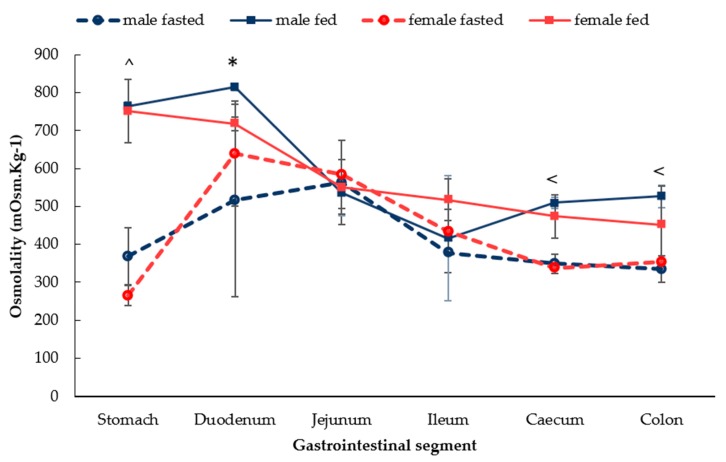
Osmolality of the GI fluids in male and female in the fasted state and fed state rats. (Mean ± SD, *n* = 6). The following symbols (i) < denotes a statistical significance (*p* < 0.05) between fasted and fed state in male rats, (ii) ^ denotes a statistical significance (*p* < 0.05) between fasted and fed state in both male and female rats and (iii) * denotes a statistical significance (*p* < 0.05) between males and females.

The osmolality of GI fluids in fasted male and female rats showed similar profiles across the whole GI tract (Figure 3). In both males and females, osmolality of the luminal fluids increased from the stomach to the proximal small intestine and reduced distally. After food intake, the osmolality of the males and females also displayed comparable trends. In both males and females, the osmolality was higher in the stomach and duodenum compared with the distal intestinal tract. In fed females, the osmolality decreased gradually from the stomach to the colon. Significant differences in the fasted and fed were observed in (i) both males and females in the stomach and caecum and (ii) in the colon of male rats. A significant sex difference was noticed in the fed duodenum, where the osmolality was 718 ± 17 mOsm.Kg^−1^ in females compared with 814 ± 10 mOsm.Kg^-1^ in males (*p* < 0.05).

#### 3.1.4. Gastrointestinal Fluid Surface Tension

**Figure 4:**
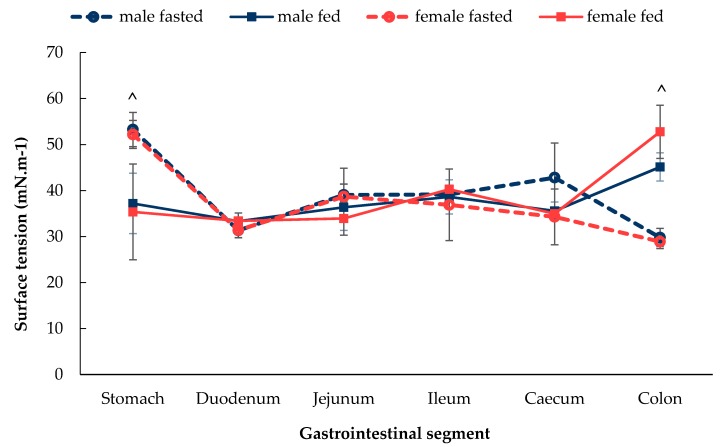
Surface tension of the fluids of sections of the GI tract of male and female in fasted state and fed state rats. (Mean ± SD, *n* = 6). The following symbol ^ denotes a statistical significance (*p* < 0.05) between fasted and fed state in both male and female rats.

As shown in Figure 4, the surface tension values of GI fluids were similar between male and female Wistar rats in the fasted and fed states along the GI tract, except for the stomach and caecum where significant differences were seen between fasted and fed male and female rats. In the fasted state of both sexes, the surface tension was the highest value in the stomach and then decreased in the duodenum and was then maintained at a lower level along the rest of the GI tract. In contrast, in the fed state, the surface tension was at a consistent level between 35–40 mN.m^−1^ from the stomach and caecum, after which it seemed to increase where the highest value was reached.

### 3.2. Sex Differences in P-gp expression Along the GI Tract

**Figure 5:**
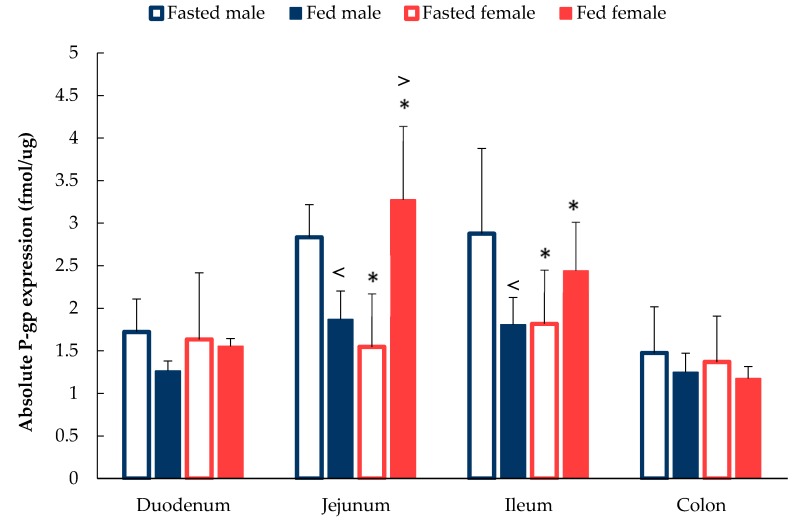
Absolute expression of P-gp along the intestinal tract in male and female rats under fasted and fed state. (Mean ± SD, *n* = 6). The following symbols (i) < denotes a statistical significance (*p* < 0.05) between fasted and fed state in male rats, (ii) > denotes a statistical significance (*p* < 0.05) between fasted and fed state in female rats and (iii) * denotes a statistical significance (*p* < 0.05) between males and females in the same state.

As shown in Figure 5, a pronounced difference was observed in the absolute expression patterns of intestinal P-gp expression between male and female rats in both the fasted and fed state. In the fasted state, there was no sex difference in both the duodenum and colon, whilst the absolute expression of intestinal P-gp showed a significant difference (*p* < 0.05) in the jejunum and ileum between male and female Wistar rats. Specifically, in the jejunum, P-gp expression in male rats was 2.83 ± 0.38 fmol/µg when compared to 1.54 ± 0.62 fmol/µg in female rats. Ileal P-gp expression followed the same trend as the jejunum where males expressed a 58% higher P-gp level than that of their female counterparts of 2.87 ± 1.00 fmol/µg versus 1.81 ± 0.63 fmol/µg, respectively. 

In contrast, a significant sex difference (*p* < 0.05) in P-gp expression was observed in rats following food intake but in the opposite direction (Figure 5). In both the jejunum and ileum, female rats expressed a much higher P-gp level than male rats, which was 3.27 ± 0.86 vs. 1.87 ± 0.33 fmol/µg and 2.44 ± 0.57 vs. 1.81 ± 0.32 fmol/µg, respectively. 

## 4. Discussion

### 4.1. Luminal Fluid Characterisation 

Following oral administration, a dosage form will be exposed to the microclimate of the subsequent parts of the GI tract, where it will undergo absorption following disintegration, dissolution, diffusion and permeation processes. It is well-known that the physicochemical properties of the luminal fluid, including pH, surface tension, buffer capacity, as well as osmolality, play an important role in these mechanisms [24,25,26,27,28].

As shown in Figure 1, Figure 2, Figure 3 and Figure 4, the luminal fluid properties are highly variable according to the location of the GI segment. Overall, no significant sex differences were identified in the pH profile expect for a lower pH in the fundus and antrum in female rats when compared to males (Figure 1). In a recent study, it was found that male rats have a higher gastric blood flow than their female counterparts [29]. Oestrogen administration was found to reduce the mean blood flow in the gastric mucosa by 31% in males; however, it remained largely unchanged in females. In addition, the gastric mucosal layer was demonstrated to thicken after oestrogen exposure at a faster rate in females than in males. This suggests that females may be more “resistant” to the administration of excess female sex hormones and, therefore, may be more effective at repairing damage to the gastric wall. If the rate of mucus production is higher in females, it may suggest an evolutionary biological adaptation to higher stomach acidity. 

The lowest pH value was expected in the stomach due to the active secretion of hydrochloric acid by parietal cells (Figure 1). The greater inter-individual variability seen in the pH in the stomach, compared to the rest of the GI tract, may due to variability in food consumption and coprophagy; the act of rats eating their own faeces or faeces from the same species [30]. The pH of the stomach is lower in the fed state due to the release of gastric acid to digest the food ‘chyme’ mixture. Although the presence of chyme in the stomach may neutralise the pH, depending on the type and quantity of food consumed and the gastric transit time. When compared with the stomach, the small intestine and colon displayed a relatively consistent pH value with low variation in both the fasted and fed state. The sharp increase in pH from the stomach to the duodenum in the fed state can be attributed to the presence of bicarbonate ions, bile and other digestive products. A slight reduction of pH in the colon and caecum was expected as the proximal large intestine is a common site for fermentation and acidic species production [31].

A study by Evans et al. described the pH profile of the human GI tract where the stomach displayed the lowest pH, which ranged from 0.4 to 4 and gradually increased from 5 in the duodenum to 7.5 in the ileum and then dropped down to 6.4 in the colon [32]. This showed a high correlation of the pH profile observed here in laboratory rats. The overall trend in the change in GI fluid pH observed in this study is similar and comparable to the pH trend in male rats and the fed-state male and female rats from previous publications [19,26,27]. 

Sex differences were observed in the duodenal buffer capacity in the fasted state (Figure 2). The buffer capacity of the luminal fluid depends on the pH, the pKa of the buffer, buffer species present and the buffer concentration [33], key parameters affecting drug solubility in the GI tract. The duodenal buffer capacity in the fasted state could be partially attributed to bile salts. The lack of a gall bladder in rodents is compensated by the enlargement of the duct system [34]. Without the storage of bile, the secreted bile acids are continually excreted into the duodenum via the duodenal papilla. A study which collected bile from 6 male and 6 female rats observed a sex difference in bile acids [35]. Female rats secrete more taurine conjugated bile acids, such as cholic acid, when compared with the male rat. Conjugated bile acids have a pKa of between 1 and 4. Therefore, in the luminal environment of the rat small intestine, where the pH is approximately 5 [26], the bile acid will exist in the ionised state. This may, in turn, contribute to the higher buffer capacity observed in the duodenum of fed female rats compared to the males. As such, the sex-related differences in bile acid composition could lead to differential solubilisation of drugs between males and females. Fed female rats displayed a higher buffer capacity (*p* < 0.05) in the caecum compared with males. This could be explained by the intake of food and possible coprophagy resulting in the production of higher quantities of buffering species by female rats in the caecum, such as short-chain fatty acids (SCFAs) [36].

A distinct variation was observed in the osmolality of GI fluids in the duodenal segment of the rats in the fasted state and a significant difference (*p* < 0.05) in the fed state. This variation may be due to the bile salt secretion, the main components of the duodenal in the luminal fluids (Figure 3). It has been reported that a variation in bile secretion in fasted rats existed [37]. A similar phenomenon was also reported in primates and humans. In primates, the independent flow of bile salt was highly variable during fasting but increased and stabilised by feeding [38]. In addition, the concentration of phospholipids in human duodenal fluids displayed a higher variation in fasted human subjects when compared to the fed-state [39]. 

The surface tension of GI fluids was significantly lower than that of water due to the presence of a multitude of compounds that act as surfactants in the GI tract, bile salts being the most well recognised (Figure 4) [40]. Due to the lack of a gall bladder in rodents, bile is released continuously into the upper duodenum of the small intestine. As a result, bile salts within the intestinal lumen reduces the surface tension of the luminal fluids. In the stomach of the fasted rate, however, the main components of the luminal fluids were water and hydrochloric acid [26], which could contribute to the observed higher surface tension compared to the rest of the GI tract. In contrast, in the fed state, the surface tension in the stomach is lower and of a similar value to the duodenum.

### 4.2. Absolute P-gp Expression

Both the changes of intestinal luminal fluids and the food–intestinal interactions during food consumption may contribute to a change in the expression of P-gp expression. The study herein quantified the absolute expression of P-gp of the intestinal segments in both male and female rats. A significant sex difference (*p* < 0.05) was reported in both the jejunal and ileal segments, which are the main sites of drug and nutrient absorption along the GI tract. The absolute P-gp expression pattern observed highly correlated to previously published studies; an in situ perfusion absorption study demonstrated that the GI absorption of verapamil, a P-gp substrate, was slightly higher in female Wistar rats (6.85 × 10^5^ ± 0.54 × 10^5^) when compared with males (6.07 × 10^5^ ± 0.58 × 10^5^) [41]. Furthermore, Dou et al. also reported that ganciclovir and ranitidine, both P-gp substrates, showed a significantly higher permeability in the jejunum and ileum of female rats than their male counterparts in the fasted state (*p* < 0.05) [21]. Moreover, the permeability of another P-gp substrate, verapamil, was higher in female rats than that in males [42]. 

An interesting phenomenon was observed in fed-state rats. In fed male rats, the absolute expression level of P-gp decreased along the whole intestinal tract when compared with fasted male rats (Figure 5). The largest reduction in P-gp intestinal expression occurred in the ileum, where levels decreased by approximately 59% from 2.88 ± 1.00 fmol/µg in fasted-state to 1.81 ± 0.19 fmol/µg in fed-state. Interestingly, in female rats, a contrasting result was observed in jejunum and ileum segments. P-gp expression increased by approximately 113% and 34% after food intake in the jejunum and ileum, respectively. 

The most intriguing results identified was the dramatic increase of P-gp in the small intestine of female rats in the fed state. In contrast to fed female rats, fed male rats displayed lower levels of P-gp expression. A suggestion is that innate protection is required for successful reproduction. Food contains multiple components, including substances which may be harmful. The body may, therefore, protect itself by increasing the expression of the efflux transporter as a barrier mechanism to prevent the absorption of potentially toxic ingested compounds.

This mechanism could be a complex interplay of the modulation of P-gp expression, enzyme reaction and the defence ability of epithelial cells, which may be further influenced by sex hormones. It was reported that oestrone and oestradiol both increase intestinal enzyme activity in female rats. By administrating 1 mg/kg oestrone and oestradiol twice a day for two days in female rats, the intestinal CYP-450 enzyme concentration increased from 0.03 ± 0.01 nmol/mg in the control group to 0.16 ± 0.01 and 0.09 ± 0.01 nmol/mg in the oestrone and oestradiol treated groups, respectively [43]. In addition, a study investigated ileum tissues obtained from both male and female rats that were exposed to harsh conditions (such as hypoxia for 40 min and acidosis at pH 6.8) and normal conditions (normoxia at a normal pH of pH 7.3) using an Ussing chamber experiment [44]. Cytokine and nitric oxide concentration levels in the Ussing chamber were subsequently measured to evaluate the immune–inflammatory response. Fluorescein Isothiocyanate-dextran (FITC-dextran, molecular weight of 4300 Da) was checked to assess the barrier function of the intestinal lumen. As a result, the female intestinal tissue showed a higher anti-inflammatory response and an enhanced intestinal barrier function when compared with males. More interestingly, the addition of oestradiol in male rats relieved the intestinal injury and enhanced their anti-inflammatory ability. 

There is a discrepancy, however, between the current study and previous studies when examining P-gp expression in the intestinal tract, in particular the colon. When quantified via Western blotting, the highest expression of P-gp is reported in the colon when compared to other segments in the GI tract [21,26]. With LC-MS/MS, however, absolute colonic P-gp expression is the lowest in the whole GI tract. Another study by Dahman and Amidon, which used immunoblotting techniques, found an increase in P-gp expression level in the distal ileum compared with the proximal jejunum of fasted male Wistar rats [45]. Our LC-MS/MS method found a similar absolute P-gp expression in the jejunum and the ileum, 2.83 ± 0.38 and 2.88 ± 1.00 fmol/µg, respectively. The possible reason for the discrepancies may lie in the principles of these two techniques. Western blotting collects target protein expression relative to the reference protein. However, a number of reference proteins for the Western blotting technique exists. When using villin, for example, colonic P-gp expression was lower than small intestinal P-gp in male rats [46]. Therefore, the lower expression of reference proteins in the colon may lead to an inflated higher calculation for the relative P-gp expression in Western blot experiments. The LC-MS/MS method used herein, however, is an absolute quantification method. A similar discrepancy was also reported in human intestinal P-gp. A study using the Western blot technique demonstrated that human intestinal P-gp relative expression gradually increased from 0.24 in duodenum to 2.13 in colon [47]. However, Drozdzik et al. reported that the absolute expression of human intestinal P-gp increased from duodenum to ileum but dropped back to the similar level of the duodenum in the colon [48]. 

Both food and sex displayed an influence on intestinal P-gp expression in rats. The data presented here show that by failing to evaluate drugs in both male and female rat models, there may be an increased risk of inaccurately extrapolating pre-clinical pharmacokinetic data into human clinical trials. As P-gp is a biological membrane efflux transporter, capable of modulating the transmembrane activities of drugs in different organs [49], this could be of particular consequence when evaluating a novel drug that is a P-gp substrate.

## 5. Conclusions

Rats have been intensively used as pre-clinical animal models in oral drug development, and, therefore, their physiology and GI tract properties have played an essential role in studies examining drug absorption. This study has illustrated that the luminal fluid properties, including pH, buffer capacity, surface tension as well as osmolality along the gastrointestinal tract of rats showed a similar pattern for male and female Wistar rats in the fasted state and fed states. Interesting, P-gp displayed a significant sex difference (*p* < 0.05) in the proximal small intestine (jejunum and ileum), the main sites of drug absorption, between male and female rats in both the fasted and fed state. The absolute quantification of P-gp expression in both male and female Wistar rats can be utilised in the design and data interpretation of pre-clinical studies for the drug development of P-gp substrates. To summarise, this current study has highlighted the sex difference of P-gp expression in the intestinal segments in the fasted- and fed-state. By better understanding the rat model, these findings can inform and improve early phase oral drug development.

## Figures and Tables

**Table 1:** Tryptic proteospecific peptide and its respective ions and mass transitions used for P-gp absolute quantification (*isotope-labelled amino acid, the labelling of Arg (R) was done by introducing C^13^ and N^15^).

Molecule Name	Peptide Sequence	Mass	Transition Number	Q1 *m/z*	Q3 *m/z*
ABCB1 (P-gp)	AGAVAEEVLAAIR (Standard)	1268.7	1	635.3	771.3
			2	635.3	900.5
			3	635.3	971.6
	AGAVAEEVLAAIR (Internal standard)	1278.6	1	640.3	781.4
			2	640.3	910.5
			3	640.3	981.5
